# Recent Antifungal Pipeline Developments against *Candida auris*: A Systematic Review

**DOI:** 10.3390/jof8111144

**Published:** 2022-10-28

**Authors:** Rogelio de J. Treviño-Rangel, Gloria M. González, Alexandra M. Montoya, Olga C. Rojas, Mariana Elizondo-Zertuche, Neri A. Álvarez-Villalobos

**Affiliations:** 1 Departamento de Microbiología, Facultad de Medicina, Universidad Autónoma de Nuevo León-Francisco I. Madero & Dr. Eduardo A. Pequeño, Mitras Centro, Monterrey 64460, Mexico; 2 Plataforma INVEST Medicina UANL-KER Unit Mayo Clinic (KER Unit Mexico), Facultad de Medicina, Universidad Autónoma de Nuevo León-Francisco I. Madero, Mitras Centro, Monterrey 64460, Mexico

**Keywords:** antifungal agents, novel therapies, *Candida auris*, systematic review

## Abstract

The alarming spread and impact of multidrug-resistant *Candida auris* infections alongside the limited therapeutic options have prompted the development of new antifungals. These promising agents are currently in different stages of development, offering novel dosing regimens and mechanisms of action. A systematic search in MEDLINE, EMBASE, Web of Science, and Scopus up to 27 June 2022 was conducted to find relevant articles reporting data of in vitro activity and in vivo efficacy of investigational antifungals against *C. auris*. These included new additions to existing antifungal classes (rezafungin and opelconazole), first-in-class drugs such as ibrexafungerp, manogepix/fosmanogepix, olorofim and tetrazoles (quilseconazole, oteseconazole and VT-1598), as well as other innovative agents like ATI-2307, MGCD290 and VL-2397. From 592 articles retrieved in the primary search, 27 met the eligibility criteria. The most studied agent was manogepix/fosmanogepix (overall MIC_90_: 0.03 mg/L), followed by ibrexafungerp (overall MIC_90_: 1 mg/L) and rezafungin (overall MIC mode: 0.25 mg/L), while VT-1598 and ATI-2307 were the least explored drugs against *C. auris*. All these compounds demonstrated significant improvements in survival and reduction in tissue fungal burden on neutropenic animal models of candidemia due to *C. auris*. Continual efforts towards the discovery of new treatments against this multidrug-resistant fungus are essential.

## 1. Introduction

*Candida auris* is a notable fungal pathogen which is an important cause of invasive infections, especially among critically ill and immunosuppressed patients, associated with mortality rates in excess of 50% [1,2]. This opportunistic yeast is resistant to some standard disinfectants, can colonize skin and mucous membranes, form biofilms, and is transmitted by contact [3]. All these attributes have led to the increased appearance of healthcare-associated outbreaks due to *C. auris* in different regions around the world. Five phylogenetically distinct clades (South Asian, East Asian, South African, South American, and Iranian) have been described based on whole-genome sequencing of clinical isolates from different continents, suggesting a simultaneous emergence [4,5].

Azoles, polyenes and echinocandins are the main classes of systemic antifungals [6]. It is well-known that these agents possess several limitations, including a narrow spectrum of activity, high toxicity, drug interactions, suboptimal pharmacokinetics, and poor bioavailability. Added to this complicated context, *C. auris* is characterized by reduced susceptibility to the three main antifungal groups [7]. Although echinocandins are recommended as first-line therapy for bloodstream *C. auris* infections [8], the emergence of pandrug-resistant *C. auris* isolates significantly compromises therapeutic options [9]. Therefore, the development and introduction of novel treatment strategies are crucial to overcome the serious challenge of resistance and to have effective agents for managing *C. auris* infections.

Conveniently, new therapeutic candidates with excellent activity against *C. auris* are in the antifungal pipeline [10]. Some developments have focused on adding new agents within existing antifungal classes, while other efforts have been aimed at improving previously approved drug formulations. Novel classes of antifungals are also being actively developed, among these are manogepix/fosmanogepix and tetrazoles. In this systematic review, we have selected twelve investigational antifungal agents for qualitative analysis and present the most recent evidence of their in vitro susceptibility and advances in in vivo studies, particularly against *C. auris*.

## 2. Methods

### 2.1. Study Design

This systematic review was conducted in accordance with the Preferred Reporting Items for Systematic Reviews and Meta-Analyses (PRISMA) guidelines [11]. Due to the basic science nature of the protocol, it was not submitted to the International Prospective Register of Systematic Reviews (PROSPERO).

### 2.2. Information Sources and Search Strategy

The search strategy was designed and executed by an experienced librarian with input from the principal investigator and the research team. A comprehensive search was conducted in MEDLINE, EMBASE, Web of Science, and Scopus electronic databases from inception to 27 June 2022 to cover all published articles relevant to our study. MeSH terms, controlled vocabulary, and keywords (File S1) were combined to search within titles and abstracts. All retrieved records were uploaded to an online software program (DistillerSR; Evidence Partners, Ottawa, ON, Canada).

### 2.3. Selection Process and Eligibility Criteria

Reviewers, working independently and in duplicate, screened studies for eligibility using standardized and prepiloted instructions in a round of title and abstract and then a full-text screen. Any disagreement was resolved by consensus or arbitration by a third reviewer. A pilot review was carried out before each phase, and the chance-adjusted agreement was quantified using the kappa statistic (k = 0.90). Original articles written exclusively in English and reporting data of in vitro susceptibility tests and animal models for therapeutic efficacy evaluation of the following antifungal agents: ibrexafungerp (IBX; SCY-078, SCY078 and MK-3118), rezafungin (RZF; CD101 and SP3025), manogepix (MGX; APX001A and E1210), fosmanogepix (FGX; APX001 and E1211), olorofim (F901318), opelconazole (OPC; PC945), quilseconazole (VT-1129), oteseconazole (VT-1161), VT-1598, ATI-2307, MGCD290 and VL-2397 tested in *C. auris* were of interest for the study and eligible. Studies that did not test the aforementioned antifungals in *C. auris* were excluded, as well as narrative and systematic reviews, opinion articles, conference posters and articles that only had the abstract available without the necessary information.

### 2.4. Data Collection

Detailed information was extracted independently and in duplicate using a standardized data extraction format, according to the type of study (in vitro susceptibility or animal model). Eligible articles were carefully reviewed, and data of interest were extracted. Basic information of each article, such as the name of the first author, country and publication date were collected regardless the type of study. When there was uncertainty regarding the results of particular articles, the corresponding authors were contacted by email for clarification.

## 3. Results

**Figure 1:**
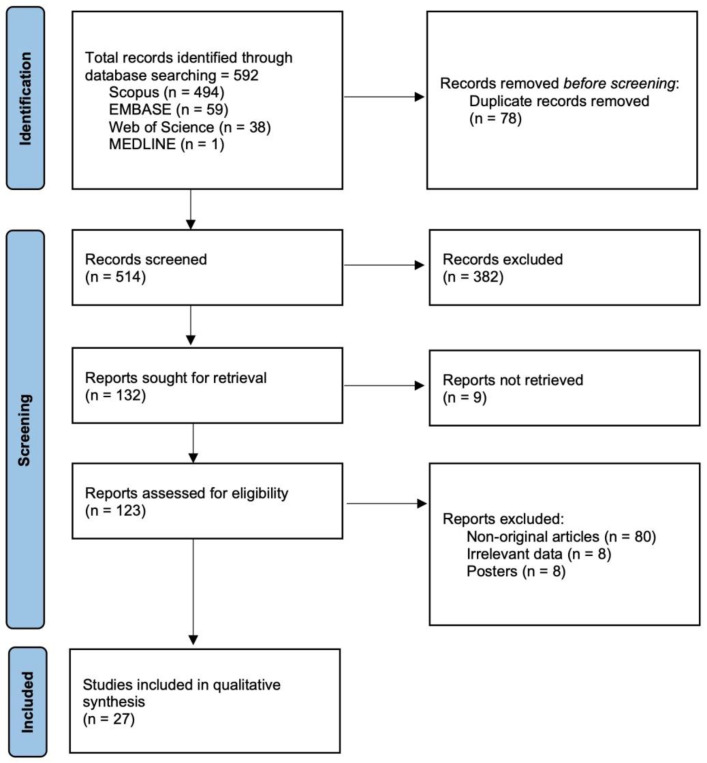
PRISMA flow diagram for selection of articles reporting data of in vitro antifungal susceptibility and therapeutic efficacy evaluation of investigational antifungals tested against *Candida auris* up to 27 June 2022.

As depicted in Figure 1, a total of 592 articles were found in the database search step, of which twenty-seven were finally eligible for inclusion in the present study. No further relevant articles were identified by cross-checking their references. The 27 studies included in this work were published between 2017 and 2022 and were conducted by renowned researchers from different countries, mainly from the USA. Additional details of the articles are shown in Table S1.

The data extracted from the articles were conveniently classified according to the type of study in two groups: (i) in vitro studies of antifungal susceptibility tests against *C. auris* isolates (Table 1), and (ii) in vivo studies of therapeutic efficacy in experimental infections due to *C. auris* (Table 2). The first group of studies originally consisted of 18 articles, while the other one just included 2. In a further revision, 7 articles were detected that evaluated the activities both in vitro and in vivo against *C. auris* of the tested drug.

The most studied investigational agent against *C. auris* was MGX/FGX with a MIC_90_ of 0.03 mg/L being the most commonly found among 10 different studies, followed by IBX (MIC_90_: 1 mg/L) and RZF (MIC mode: 0.25 mg/L) with 7 studies each. VT-1598 (MIC_90_: 1 mg/L) and ATI-2307 (MIC_90_: 0.015 mg/L) were the least explored drugs against *C. auris*. All these compounds demonstrated significant improvements in survival and reductions in tissue fungal burden (FB) in neutropenic murine models of disseminated infection caused by *C. auris*. At the moment of the search, no studies were found that evaluated the activity of olorofim (lacks in vitro activity against yeasts [12]), quilseconazole, oteseconazole, MGCD290 or VL-2397 against *C. auris*.

**Table 1:** Studies of in vitro antifungal susceptibility of investigational antifungals against *Candida auris*.

					Susceptibility Results					Ref.
Study		*C. auris* Isolates Evaluated			Methodology	MIC (mg/L)				
First Author	Year	*n*	Origin	Clades *^a^*		MIC_50_	MIC_90_	Range	GM (Mode)	
Ibrexafungerp										
Berkow	2017	100	NS	NS	CLSI	0.5	1	0.06–2	(1)	[13]
Larkin	2017	16	Blood (15), ear (1)	NS	CLSI	1	1	0.5–2	NS	[14]
Zhu	2020	195	NS	NS	CLSI	NS	NS	0.06–8	0.407 (0.5)	[15]
Arendrup	2020	122	Blood (100), miscellaneous (22)	NS	EUCAST	0.5	NS	0.06–2	(0.5)	[16]
Wiederhold	2021	54	NS	NS	CLSI	1	1	0.25–2	0.764	[17]
Quindós	2022	22	Blood (8), oral specimens (7), urine (7)	NS	EUCAST	0.5	2	0.5–8	0.753 (0.5)	[18]
Rezafungin										
Berkow	2018	100	NS	NS	CLSI	0.125	0.5	0.03–8	(0.25)	[19]
Lepak	2018	4	NS	NS	CLSI	-	-	0.06–2	-	[20]
Tóth	2019	19	NS	NS	CLSI	0.125	0.25	0.03–0.25	(0.125–0.25)	[21]
Helleberg	2020	122	Blood (100), miscellaneous (22)	NS	EUCAST	0.25	1	0.06–16	NS	[22]
Tóth	2020	16	NS	Clade I (8), clade II (2), clade III (6)	CLSI	NS	NS	0.25–1	NS	[23]
Kovács	2021	13	Blood (4), miscellaneous (7), environmental (2)	Clade I (3), clade II (3), clade III (3), clade IV (4)	CLSI	NS	NS	0.03–0.25	NS	[24]
Manogepix										
Arendrup	2018	122	Blood (100), miscellaneous (22)	NS	EUCAST	0.016	0.03	0.001–0.125	(0.016)	[25]
Berkow	2018	100	NS	NS	CLSI	0.002	0.008	<0.005–0.015	(<0.005)	[26]
Hager	2018	16	Blood (15), ear (1)	NS	CLSI	0.004	0.03	0.002–0.06	NS	[27]
Zhao	2018	4	NS	NS	CLSI	-	-	0.004–0.03	-	[28]
Wiederhold	2019	13	NS	NS	CLSI	0.03	0.125	≤0.002–0.03	0.013	[29]
Pfaller	2019	1	NS	NS	CLSI	-	-	0.06	-	[30]
Zhu	2020	200	Blood (42), urine (36), nares (21), miscellaneous (83), environmental (18)	NS	CLSI	0.03	0.03	0.004–0.06	0.02 (0.03)	[31]
Arendrup	2020	122	Blood (100), miscellaneous (22)	NS	CLSI	0.008	0.03	0.001–0.25	0.01	[32]
Pfaller	2021	11	NS	Clade I (5), clade IV (6)	CLSI	0.015	0.03	≤0.002–0.06	NS	[33]
Maphanga	2022	394	Blood	Clade I (13), clade III (70), clade IV (1)	CLSI	0.008	0.016	0.002–0.06	0.008 (0.008)	[34]
Opelconazole										
Rudramurthy	2019	72	NS	NS	CLSI	0.06 *^b^*	0.25 *^b^*	NS	0.06 (0.06) *^b^*	[35]
VT-1598										
Wiederhold	2019	100	NS	Clade I (47), clade II (3), clade III (11), clade IV (39)	CLSI	0.25	1	0.03–8	(0.25)	[36]
ATI-2307										
Wiederhold	2020	23	NS	Clade I, clade IV	CLSI	0.015 *^c^*	0.015 *^c^*	≤0.008–0.015 *^c^*	0.011 *^c^*	[37]

*^a^* Clades: clade I (South Asia), clade II (East Asia), clade III (South Africa), clade IV (South America). *^b^* Data obtained from the 24 h MIC readings. *^c^* Data obtained from the 24 h MIC readings (50% inhibition). NS: not specified; MIC: minimum inhibitory concentration; GM: geometric mean; CLSI: clinical & laboratory standards institute; EUCAST: European committee on antimicrobial susceptibility testing.

**Table 2:** In vivo studies of investigational antifungals tested in experimental infections due to *C. auris*.

Study		Animal Model							Ref.
First Author	Year	*C. auris* Isolate (Origin, Country)	Model	Immunosupression Regimen & Administration	Infection Route	[Inoculum]	Antifungal Posology & Administration	Relevant Findings	
Ibrexafungerp									
Ghannoum	2020	MRL 35368	Guinea pig	Prednisolone 30 mg/kg (days −1, +1 and +3), s.q.	cut.	1 × 10^9^ CFU/mL	10, 20 and 30 mg/kg, p.o.	The dose of 10 mg/kg reduced severity of lesions and significantly reduced FB (average log_10_ CFU/g: 2.8) vs. untreated control	[38]
Wiederhold	2021	UTHSCSA DI17–46 (blood, USA)	Male ICR mice	5-fluorouracil 5 mg/mouse (day −1), i.v.	i.v.	1 × 10^7^ cells/mouse (survival arm) 5 × 10^6^ cells/mouse (FB arm)	20, 30 and 40 mg/kg BID for 7 days, p.o.	>60% survival, reductions in kidney FB (average log_10_ CFU/g: 1.83–3.85) vs. untreated control	[17]
Rezafungin									
Lepak	2018	B11220 (Japan), B11785 (Colombia), B11799 (Colombia), B11211 (India)	Mice	Cyclophosphamide 150 mg/kg (day −4) and 100 mg/kg (days −1, +2 and +4), s.q.	i.v.	5.99 ± 0.29 log_10_ CFU/mL	1, 4, 16 and 64 mg/kg q 3rd day for 7 days, i.p.	Stasis free-drug 24 h AUC/MIC target: 1.88 1-log-kill free-drug 24 h AUC/MIC target: 5.77	[20]
Hager	2018	MRL 35368	Female CD-1 mice	Cyclophosphamide 200 mg/kg (day −3) and 150 mg/kg (day +1), i.p.	i.v.	3 × 10^7^ blastospores/0.1 mL	20 mg/kg days +1, +3 and +6, i.p.	Significantly lower FB in kidney vs. untreated control on all time points (~4 log_10_ CFU/g for day +10)	[39]
Fosmanogepix									
Hager	2018	CBS 12766 (blood, India)	Female CD1 mice	Cyclophosphamide 200 mg/kg (day −3) and 150 mg/kg (day +1), i.p.	i.v.	3 × 10^7^ blastospores/0.1 mL	78 mg/kg BID, 78 mg/kg TID and 104 mg/kg BID, i.p.	80–100% survival in all groups, significant FB reduction (1.03–1.83 log_10_ CFU/g) in kidney, lung and brain vs. untreated control	[27]
Zhao	2018	B11104, B11221, B11219, B11804 (C54007)	Female ICR/Swiss mice	Cyclophosphamide 150 mg/kg (day −4), 100 mg/kg (day −1) and 100 mg/kg (day +2), s.q.	i.v.	6.30 ± 0.07 log_10_ CFU/mL	1–256 mg/kg q 6 h for 96 h, p.o.	ED_50_: 7.14 ± 4.54 Stasis *f*AUC/MIC target: 14.67 ± 8.30	[28]
Wiederhold	2019	UTHSCSA DI17–46 (blood, USA)	Mice	5-fluorouracil 5 mg (day −1), i.v.	i.v.	1 × 10^7^ cells/mouse (survival arm) 5 × 10^6^ cells/mouse (FB arm)	104 mg/kg TID, 130 mg/kg TID and 260 mg/kg BID, for 7 days, i.p.	90–100% survival, reductions in FB in kidney (3.86 log_10_ CFU/g) and brain (2.99 log_10_ CFU/g) vs. untreated control with the highest dose in FB arm and in each group of survival arm	[29]
VT-1598									
Wiederhold	2019	UTHSCSA DI17–46 (blood, USA)	Mice	5-fluorouracil 5 mg/mouse (day −1), i.v.	i.v.	1 × 10^7^ cells/mouse (survival arm) 5 × 10^6^ cells/mouse (FB arm)	5, 15 and 50 mg/kg for 7 days, p.o.	Significant and dose-dependent improvements in survival (90%), reductions in kidney and brain FB (1.88–3.61 log_10_ CFU/g) vs. untreated control	[36]
ATI-2307									
Wiederhold	2020	UTHSCSA DI17–46 (blood, USA)	Male ICR mice	5-fluorouracil 5 mg/mouse (day −1), i.v.	i.v.	1 × 10^7^ cells/mouse (survival arm) 5 × 10^6^ cells/mouse (FB arm)	0.75, 1.5 and 3 mg/kg for 7 days, s.c.	The dose of 3 mg/kg significantly improved survival (70%) and reduced kidney FB (5.06 log_10_ CFU/g) vs. untreated control	[37]

i.p.: intraperitoneal; s.q.: subcutaneous; i.v.: intravenous; p.o.: oral (oral gavage); cut.: cutaneous; BID: twice daily; TID: three times daily; FB: fungal burden; CFU: colony forming units; ED_50_: dose required to achieve 50% of the maximum effect; AUC: area under the curve.

## 4. Discussion

Antifungal resistance is a growing threat that presents a major clinical challenge in the treatment of *C. auris* infections due to the multidrug-resistant profile of this remarkable fungus. Hence, there is a critical need to expand our antifungal armamentarium and develop new effective agents with novel fungal targets, low toxicity, and preferably high oral bioavailability. Nevertheless, there is hope on the horizon, the antifungal pipeline has finally responded with some promising prospects for managing these deadly infections.

Investigational agents currently in phase III clinical trials are IBX and RZF. IBX (Scynexis, Inc.; Jersey City, NJ, USA) is a semi-synthetic derivative from the natural product enfumafungin, which represents the first of the triterpenoid antifungals [40]. Like echinocandins, it inhibits 1,3-β-ᴅ-glucan synthase but via alternative binding sites resulting in limited cross-resistance with conventional candins [41]. IBX is highly bioavailable and was developed for both oral and intravenous administration [40], but only the former formulation has been studied in humans to date. The in vitro activity of this investigational agent against *C. auris* has been studied since 2017 (Table 1) [13,14,15,16,17,18]. Berkow et al. [13] examined the in vitro susceptibility of IBX against a collection of 100 isolates of *C. auris*, which included each of the original four phylogenetic clades and originated from various countries, including India, Pakistan, Colombia, South Africa, and the United States. They reported an excellent antifungal activity of IBX (MIC mode and MIC_90_ of 1 mg/L), without significant differences among MIC values between the clades. Larkin et al. [14] also reported similar findings in a small subset of 16 clinical isolates of *C. auris*, mainly from bloodstream. Additionally, they demonstrated that *C. auris* biofilms treated with IBX exhibit reduced metabolic activity and thickness in comparison with untreated control biofilms [14]. Later, Zhu et al. [15] informed that amongst 195 isolates of *C. auris* from an outbreak in New York State, USA, 194 were susceptible to IBX with a mean MIC of 0.407 mg/L, including 5 pan-resistant isolates. Interestingly, they found one isolate with an IBX MIC of 8 mg/L. More recently, Arendrup et al. [16] and Quindós et al. [18] evaluated the in vitro activity of IBX following the EUCAST protocol in a collection of 122 and 22 *C. auris* isolates, respectively. Both studies found similar results with a MIC mode and MIC_50_ of 0.5 mg/L. In parallel, in vivo studies evaluating the efficacy of IBX against *C. auris* have also been carried out (Table 2). This agent has been shown to improve survival and decrease tissue FB in an immunosuppressed mouse [17] and guinea pig [38] models of *C. auris* infections. An open-label phase III study (CARES; NCT03363841) is currently underway in India and the US to evaluate the safety and efficacy of IBX for *C. auris* infection is ongoing and 2 cases of *C. auris* candidemia successfully treated with oral IBX have been preliminary reported [42].

RZF (Cidara Therapeutics; San Diego, CA, USA) is a novel echinocandin with enhanced PK/PD pharmacometrics, which is structurally similar to anidulafungin. Chemical modifications have increased its stability and solubility, granting a prolonged half-life that allows for intravenous dosing once weekly [43]. As depicted in Table 1, there are some reports showing encouraging in vitro activity of RZF against *C. auris* [19,20,21,22,23,24]. Berkow et al. [19] described the in vitro susceptibilities of a large collection of 100 *C. auris* isolates employing the CLSI methodology and reported a good antifungal activity with a MIC_50_ and MIC_90_ of 0.125 mg/L and 0.5 mg/L, respectively. Interestingly, they found 4 isolates exhibiting high RZF MICs harboring the S639P substitution in FKS1 hot spot 1. Helleberg et al. [22] communicated similar results later following the EUCAST protocol. They tested RZF in 122 clinical isolates of *C. auris* from India and informed that ~15% were non-wild type isolates and 8 harbored the S639F substitution in Fks1 hot spot, displaying MICs of 8–16 mg/L. In addition, trailing effect has been recently reported for this next-generation candin when tested in *C. auris* isolates [23]. On the other hand, RZF has also been evaluated in vivo, particularly in models of invasive candidiasis in neutropenic mice (Table 2). Lepak et al. [20] estimated a MIC ceiling of 1–2 mg/L to achieve 1-log-kill target exposures against *C. auris*, and of 2–4 mg/L for the stasis target. Importantly, there have been reported *C. auris* isolates exhibiting MICs that are at or above the aforementioned estimated MIC ceilings, as mentioned in the previous studies [19,22]. Moreover, the in vivo therapeutic efficacy of RZF was superior in terms of tissue FB reduction compared to micafungin and amphotericin B in an experimentally induced disseminated *C. auris* infection [39].

Other drugs, like FGX and OPC, are now in phase II clinical trials. FGX (Amplyx Pharmaceuticals, Inc. [recently acquired by Pfizer, Inc.]; San Diego, CA, USA) is a prodrug that is rapidly hydrolyzed by systemic phosphatases to the active moiety MGX following oral or intravenous administration. This first-in-class broad spectrum agent inhibits Gwt1, a fungal-specific enzyme involved in the glycosylphosphatidylinositol (GPI)-anchor biosynthesis, compromising cell wall integrity and restricting fungal growth [44]. MGX is a highly active compound against *C. auris* [25,26,27,28,29,30,31,32,33,34], with an overall MIC_90_ of 0.03 mg/L (Table 1). Arendrup et al. [25] reported a MGX MIC range of 0.001–0.125 mg/L and a wild-type upper limit (WT-UL) of 0.016 mg/L for a collection of 122 Indian *C. auris* isolates evaluated by the EUCAST methodology. The same research group then determined the in vitro susceptibilities of these isolates to MGX following the CLSI protocol in order to compare head-to-head the MIC data sets, encountering an excellent essential agreement between both reference methodologies [32]. One of the largest collections of *C. auris* isolates evaluated for MGX susceptibility was that tested by Zhu et al. [31]. Among 200 isolates from the New York outbreak recovered from diverse origins, they reported a MGX MIC range of 0.004–0.06 mg/L, which is within two dilutions of the ranges reported by other authors [26,27,33,34]. In addition, they did not find non-WT *C. auris* isolates for MGX at the WT-UL of 0.06 mg/L and MGX retained potent in vitro activity against pandrug-resistant *C. auris* isolates, agreeing with Maphanga et al. [34]. It is worth mentioning that while Arendrup et al. [32] found a positive correlation between fluconazole and MGX MICs in those isolates, Maphanga et al. [34] did not encounter any difference in MGX activity among isolates resistant or susceptible to fluconazole. The FGX PK/PD was recently assessed in an immunosuppressed murine model of candidemia, in which concentration-dependent efficacy against *C. auris* was demonstrated [28]. Furthermore, in a similar animal model, treatment with FGX led to an increased survival and decreased brain FB compared to anidulafungin [27]. Likewise, FGX exhibited significant improvements in survival and kidney FB reductions in a delayed therapy model of invasive candidiasis caused by *C. auris* [29]. An open-label phase II study (APEX, NCT04148287) addressing the safety and efficacy of FGX for the treatment of candidemia and/or invasive candidiasis due to *C. auris* has just been completed. In this trial, FGX therapy was instituted for up to 42 days, with a follow-up of 4 weeks after study completion. Otherwise, OPC (Pulmocide Ltd.; London, UK) is the first broad-spectrum triazole designed and optimized for once-daily, topical or inhaled therapy for mycoses of the sinopulmonary tract [45]. Its mechanism of action is familiar and comparable to established azoles. Among a global collection of 72 clinical isolates of *C. auris*, OPC (MIC_90_: 0.25 mg/L) exhibited 7.4-fold and 1.5-fold more potent in vitro activity than voriconazole and posaconazole, respectively [35]. This new triazole is under clinical development (NCT02715570).

In addition to the above-mentioned agents being at the stage of phase III/II clinical trials, other promising compounds are contemplated for future clinical application for *C. auris* infections. These include the fungal Cyp51-specific inhibitor VT-1598 and the mitochondrial respiratory chain inhibitor ATI-2307. VT-1598 (Mycovia Pharmaceuticals, Inc.; Durham, NC, USA) is part of the next-generation of oral azoles, in which the characteristic 1-(1,2,4-triazole) metal-binding group has been replaced with a tetrazole [46]. It demonstrated in vitro activity against a large collection of 100 *C. auris* isolates (MIC mode: 0.25 mg/L), which also translated into in vivo efficacy in a neutropenic murine model of candidemia significantly improving survival and reducing tissue FB in a dose-dependent manner compared to control [36]. This investigational agent is currently in phase I trials for potential use in *C. auris* infections (NCT04208321). On the other hand, ATI-2307 (Toyamara Chemical Co. [recently acquired by Appili Therapeutics, Inc.; Halifax, NS, Canada]) is a first-in-class arylamidine that selectively causes the collapse of fungal mitochondrial membrane potential [47], it is only available as a parenteral formulation at present. ATI-2307 exhibited in vitro activity against *C. auris* (MIC ranges: ≤0.008–0.015 mg/L), as well as in vivo efficacy in a mouse model of disseminated infection caused by the same fungal pathogen [37]. Of note, other novel compounds, MYC-053 and SCY-247, with potent in vitro activity against *C. auris* have recently been developed [48,49].

Overall, among the investigational agents reviewed in this study, MGX exhibited the most potent in vitro activity against *C. auris*, which was also corroborated in vivo with a considerable survival advantage and significant reductions in tissue FB. Although differences in antifungal efficacy against *C. auris* are evident between all these compounds, the different designs and methodology of the analyzed studies make it unreasonable to establish direct comparisons. Further studies must be carried out in order to verify its effectiveness in the clinical setting.

## 5. Conclusions

We systematically reviewed investigational antifungals in clinical phases of development with activity against *C. auris*, including two agents within two novel antifungal classes targeting the fungal cell wall, IBX and FGX; both have a selective advantage as oral fungicidal therapy for *C. auris*. Additionally, two other compounds within existing antifungal classes, RZF and OPC, demonstrate enhanced PK/PD properties that, importantly, afford improved safety and tolerability profiles. Moreover, VT-1598 and ATI-2307 are first-in-class fungal specific inhibitors that have recently shown potent in vitro and in vivo activity against *C. auris*. All these agents are a very welcome addition to replenishing the antifungal arsenal, so that the results of ongoing clinical trials are eagerly awaited.

## Data Availability

The data of this systematic review are available in the presented manuscript.

## Supplementary Materials

The following supporting information can be downloaded at: https://www.mdpi.com/article/10.3390/jof8111144/s1, File S1: Search strategy, Table S1: Basic characteristics of the articles included in this systematic review.
