# Supplementary material for: Recent Antifungal Pipeline Developments against Candida auris: A Systematic Review
Source: J Fungi (Basel). 2022 Oct 28;8(11):1144. doi: 10.3390/jof8111144 (PMC9695453; doi:10.3390/jof8111144)
Supplement: Supplementary file 1 [file jof-08-01144-s001.zip › jof-1945909-supplementary.pdf]

File S1. Search strategy.

SCOPUS: June 27, 2022

((*rezafungin* OR *"1 [4 hydroxy N2 [[4'' (pentyloxy)[1,1':4',1'' terphenyl] 4 yl]carbonyl] 5 [2 (trimethylammonio)ethoxy]ornithine] 4 [4 hydroxy 4 (4 hydroxyphenyl)allothreonine] echinocandin B "* OR *"2 [[23 [1,2 dihydroxy 2 (4 hydroxyphenyl)ethyl] 2,11,15 trihydroxy 6,20 bis(1 hydroxyethyl) 16 methyl 5,8,14,19,22,25 hexaoxo 9 [[[4'' (pentyloxy) 1,1':4',1'' terphenyl 4 yl]carbonyl]amino]tetracosahydro 1h dipyrrolo[2,1–c:2',1'–l][1,4,7,10,13,16]hexaazacyclohenicosin 12 yl]oxy] n,n,n trimethylethanaminium "* OR *biafungin* OR *"biafungin acetate "* OR *"cd 101 "* OR *"cd101"* OR *"rezafungin acetate"* OR *"sp 3025"* OR *"sp3025"* OR *ibrexafungerp* OR *"15 (2 amino 2,3,3 trimethylbutoxy) 1,6a,8,10a tetrameth yl 8 (3 methyl 2 butanyl) 14 [5 (4 pyridinyl) 1h 1,2,4 triazol 1 yl] 1,6,6a,7,8,9,10,10a,10b,11,12,12a dodecahydro 2h,4h 1,4a propanophenanthro[1,2 c]pyran 7 carboxylic acid 2 hydroxy 1,2,3 propanetricarboxylate "* OR *"15 (2 amino 2,3,3 trimethylbutoxy) 1,6a,8,10a tetrameth yl 8 (3 methyl 2 butanyl) 14 [5 (4 pyridinyl) 1h 1,2,4 triazol 1 yl] 1,6,6a,7,8,9,10,10a,10b,11,12,12a dodecahydro 2h,4h 1,4a propanophenanthro[1,2 c]pyran7 carboxylic acid"* OR *"15 (2 amino 2,3,3 trimethylbutoxy) 1,6a,8,10a tetrameth yl 8 (3 methylbutan 2 yl) 14 [5 (pyridin 4 yl) 1h 1,2,4 triazol 1 yl] 1,6,6a,7,8,9,10,10a,10b,11,12,12a dodecahydro 2h,4h 1,4a propanophenanthro[1,2 c]pyran 7 carboxylic acid 2 hydroxy 1,2,3 propanetricarboxylate"* OR *"15 (2 amino 2,3,3 trimethylbutoxy) 1,6a,8,10a tetrameth yl 8 (3 methylbutan 2 yl) 14 [5 (pyridin 4 yl) 1h 1,2,4 triazol 1 yl] 1,6,6a,7,8,9,10,10a,10b,11,12,12a dodecahydro 2h,4h 1,4a propanophenanthro[1,2 c]pyran7 carboxylic acid"* OR *"2 (2 amino 2,3,3 trimethylbutoxy) 1,6a,8,10a tetramethyl 8 (3 methyl 2 butanyl) 3 [5 (4 pyridinyl) 1h 1,2,4 triazol 1 yl] 1,3,4,6,6a,7,8,9,10,10a,10b,11,12,12a tetradecahydro 2h 1,4a (methanooxymethano)chrysene 7 carboxylic acid"* OR *"2 (2 amino 2,3,3 trimethylbutoxy) 1,6a,8,10a tetramethyl 8 (3 methyl 2 butanyl) 3 [5 (4 pyridinyl) 1h 1,2,4 triazol 1 yl] 1,3,4,6,6a,7,8,9,10,10a,10b,11,12,12a tetradecahydro 2h 1,4a (methanooxymethano)chrysene 7 carboxylic acid 2 hydroxy 1,2,3 propanetricarboxylate"* OR *"2 (2 amino 2,3,3 trimethylbutoxy) 1,6a,8,10a tetramethyl 8 (3 methylbutan 2 yl) 3 [5 (pyridin 4 yl) 1h 1,2,4 triazol 1 yl] 1,3,4,6,6a,7,8,9,10,10a,10b,11,12,12a tetradecahydro 2h 1,4a (methanooxymethano)chrysene 7 carboxylic acid"* OR *"2 (2 amino 2,3,3 trimethylbutoxy) 1,6a,8,10a tetramethyl 8 (3 methylbutan 2 yl) 3 [5 (pyridin 4 yl) 1h 1,2,4 triazol 1 yl] 1,3,4,6,6a,7,8,9,10,10a,10b,11,12,12a tetradecahydro 2h 1,4a (methanooxymethano)chrysene 7 carboxylic acid 2 hydroxy 1,2,3 propanetricarboxylate"* OR *"ibrexafungerp citrate"* OR *"mk 3118"* OR *"mk3118"* OR *"scy 078"* OR *"scy 78"* OR *"scy078"* OR *"scy78"* OR *olorofim* OR *"2 (1,5 dimethyl 3 phenyl 1h pyrrol 2 yl) n [4 [4 (5 fluoro 2 pyrimidinyl) 1 piperazinyl]phenyl] 2 oxoacetamide"* OR *"2 (1,5 dimethyl 3 phenyl 1h pyrrol 2 yl) n [4 [4 (5 fluoropyrimidin 2 yl)piperazin 1 yl]phenyl] 2 oxoacetamide"* OR *"f 901318"* OR *"f901318"* OR *fosmanogepix* OR *" (12 amino 6 oxa 1(3),7(2) dipyridina 2(5,3) (1,2)oxazola 4(1,4) benzenaheptaphan 11 ium 11 yl)methyl hydrogen phosphate"* OR *"2 amino 1 [(phosphonooxy)methyl] 3 [3 [[4 [(2 pyridinyloxy)methyl]phenyl]methyl] 5 isoxazolyl]pyridinium"* OR *"2 amino 1 [(phosphonooxy)methyl] 3 [3 [[4 [(pyridin 2 yloxy)methyl]phenyl]methyl] 5 isoxazolyl]pyridinium"* OR *" [2 amino 3 [3 [[4 (2 pyridinyloxymethyl)phenyl]methyl] 5 isoxazolyl]pyridin 1 ium 1 yl]methyl hydrogen phosphate"* OR *" [2 amino 3 [3 [[4 (pyridin 2 yloxymethyl)phenyl]methyl] 1,2 oxazol 5 yl]pyridin 1 ium 1 yl]methyl hydrogen phosphate"* OR *"apx 001"* OR *"apx001"* OR *"e 1211"* OR *"e1211"* OR *quilseconazole* OR *"2 (2,4 difluorophenyl) 1,1 difluoro 3 (1h tetrazol 1 yl) 1 [5 [4 (trifluoromethoxy)phenyl] 2 pyridinyl] 2 propanol"* OR *"2 (2,4 difluorophenyl) 1,1 difluoro 3 (1h tetrazol 1 yl) 1 [5 [4 (trifluoromethoxy)phenyl]pyridin 2 yl]propan 2 ol"* OR *"alpha (2,4 difluorophenyl) beta,beta difluoro alpha (1h tetrazol 1 ylmethyl) 5 [4 (trifluoromethoxy)phenyl] 2 pyridineethanol"* OR *"vt 1129"* OR *"vt1129"* OR *oteseconazole* OR *"1 (2,4 difluorophenyl) 2,2 difluoro 1 [(1h 1,2,3,4 tetrazol 1*

yl)methyl] 2 [5 [4 (2,2,2 trifluoroethoxy)phenyl] 2 pyridinyl]ethanol" OR *"1 (2,4 difluorophenyl) 2,2 difluoro 1 [(1h 1,2,3,4 tetrazol 1 yl)methyl] 2 [5 [4 (2,2,2 trifluoroethoxy)phenyl]pyridin 2 yl]ethanol"* OR *"2 (2,4 difluorophenyl) 1,1 difluoro 3 (1h 1,2,3,4 tetrazol 1 yl) 1 [5 [4 (2,2,2 trifluoroethoxy)phenyl] 2 pyridinyl] 2 propanol"* OR *"2 (2,4 difluorophenyl) 1,1 difluoro 3 (1h 1,2,3,4 tetrazol 1 yl) 1 [5 [4 (2,2,2 trifluoroethoxy)phenyl]pyridin 2 yl]propan 2 ol"* OR *"alpha (2,4 difluorophenyl) beta,beta difluoro alpha (1h tetrazol 1 ylmethyl) 5 [4 (2,2,2 trifluoroethoxy)phenyl] 2 pyridineethanol"* OR *"vt 1161"* OR *"vt1161"* OR *opelconazole* OR *"4 [4 [4 [[5 [(1,2,4 triazol 1 yl)methyl] 5 (2,4 difluorophenyl)tetrahydrofuran 3 yl]methoxy] 3 methylphenyl]piperazin 1 yl] n (4 fluorophenyl)benzamide"* OR *"4 [4 [4 [[5 [(1h 1,2,4 triazol 1 yl)methyl] 5 (2,4 difluorophenyl)tetrahydrofuran 3 yl]methoxy] 3 methylphenyl]piperazin 1 yl] n (4 fluorophenyl)benzamide"* OR *"6(2) (2,4 difluorophenyl) n (4 fluorophenyl) 3(3) methyl 4 oxa 2(1,4) piperazina 8(1) [1,2,4]triazola 6(4,2) oxolana 1(1),3(1,4) dibenzenaoctaphane 1(4) carboxamide"* OR *"pc 945"* OR *"pc945"* OR *"T-2307"* OR *"ATI-2307"* OR *"MGCD290"* OR *"VL-2397"*) AND *"Candida auris"*

Table S1. Basic characteristics of the articles included in this systematic review.

First author	Year of publication	Country	Agent evaluated	Type of study	Ref.
Arendrup	2018	Denmark	Manogepix	*In vitro*	32
Arendrup	2020	Denmark	Manogepix	*In vitro*	39
Arendrup	2020	Denmark	Ibrexafungerp	*In vitro*	18
Berkow	2017	USA	Ibrexafungerp	*In vitro*	15
Berkow	2018	USA	Rezafungin	*In vitro*	24
Berkow	2018	USA	Manogepix	*In vitro*	33
Ghannoum	2020	USA	Ibrexafungerp	Animal model	21
Hager	2018	USA	Manogepix/fosmanogepix	*In vitro*/animal model	34
Hager	2018	USA	Rezafungin	Animal model	30
Helleberg	2020	Denmark	Rezafungin	*In vitro*	27
Kovács	2021	Hungary	Rezafungin	*In vitro*	29
Larkin	2017	USA	Ibrexafungerp	*In vitro*	16
Lepak	2018	USA	Rezafungin	*In vitro*/animal model	25
Maphanga	2022	South Africa	Manogepix	*In vitro*	41
Pfaller	2019	USA	Manogepix	*In vitro*	37
Pfaller	2021	USA	Manogepix	*In vitro*	40
Quindós	2022	Spain	Ibrexafungerp	*In vitro*	20
Rudramurthy	2019	India	Opelconazole	*In vitro*	43
Tóth	2020	Hungary	Rezafungin	*In vitro*	28
Tóth	2019	Hungary	Rezafungin	*In vitro*	26
Wiederhold	2019	USA	VT-1598	*In vitro*/animal model	45
Wiederhold	2020	USA	ATI-2307	*In vitro*/animal model	47
Wiederhold	2021	USA	Ibrexafungerp	*In vitro*/animal model	19
Wiederhold	2019	USA	Fosmanogepix	*In vitro*/animal model	36
Zhao	2018	USA	Fosmanogepix	*In vitro*/animal model	35
Zhu	2020	USA	Manogepix	*In vitro*	38
Zhu	2020	USA	Ibrexafungerp	*In vitro*	17
